# Prevalence des cardiopathies infantiles symptomatiques au Centre Hospitalier Régional de Louga, Senegal

**DOI:** 10.5830/CVJA-2015-031

**Published:** 2015

**Authors:** Georges Antoine Bazolo Ba Ngouala, Désiré Alain Affangla, Mohamed Leye, Abdoul Kane

**Affiliations:** Service de pédiatrie, Centre Hospitalier Régional de Louga, Louga, Sénégal; Service de cardiologie, Hôpital Saint Jean de Dieu, Thiès, Sénégal; Service de médecine, Hôpital Barthimée, Thiès, Sénégal; Service de cardiologie, Hôpital Général Grand Yoff, Dakar, Sénégal

**Keywords:** cardiopathie congénitale, cardiopathies acquises, chirurgie cardiaque, association humanitaire, Afrique, congenital heart disease, acquired heart disease, cardiac surgery, humanitarian association, Africa

## Abstract

La prise en charge des cardiopathies infantiles congénitales ou acquises dans les pays d’Afrique au sud du Sahara posent encore d’énormes difficultés de diagnostic et d’accès au traitement notamment chirurgical. Les objectifs de ce travail rétrospectif étaient de déterminer la prévalence des cardiopathies observées en milieu pédiatrique au Centre Hospitalier Régional (CHR) de Louga, de décrire les différents types observés et de rapporter leur évolution à court terme.

Durant la période d’étude, 1 Juillet 2009 au 31 Décembre 2012, 82 enfants sur 18 815 enfants présentaient une cardiopathie, soit une prévalence de 4.3/1 000. On note une prédominance du sexe féminin avec un sexe ratio de 1.2. Les circonstances de découverte les plus fréquentes sont représentées par la dyspnée 47.5% suivie du souffle cardiaque 35.3% et de l’insuffisance cardiaque congestive 13.4%. Les cardiopathies congénitales sont les plus fréquentes avec 69.5% des cas suivi des cardiopathies acquises avec 29.3% des cas et des formes mixtes avec 1.2%. Les principales cardiopathies congénitales retrouvées sont la communication inter ventriculaire (24.3%) suivie des canaux atrio-ventriculaires (12.1%), de la tétralogie de Fallot (9.7%) et de la persistance du canal artériel (7.3%). Les cardiopathies rhumatismales retrouvées dans 25.6% des cas et les péricardites tuberculeuses dans 3.7% des cas représentent les formes acquises. La mortalité est élevée avec 20 enfants décédés (24.4%) pendant la période d’étude. Seuls 13 patients sur 82 (15.9%) présentant une indication opératoire ont été opérés en France grâce à une prise en charge par l’association humanitaire Mécénat Chirurgie Cardiaque.

Ainsi donc les cardiopathies infantiles sont peu fréquentes dans le service de pédiatrie du CHR de Louga. Les formes congénitales sont plus fréquentes que les formes acquises. Leur mortalité est élevée et l’accès à la chirurgie reste faible.

## Abstract (en)

The management of congenital or acquired infantile heart diseases in sub-Saharan African countries still presents problems, particularly with diagnosis and access to surgical treatment. Our objectives were to describe the heart diseases observed in the paediatric setting of the Louga Regional Hospital (LRH) and report their short-term evolution.

In the study period from 1 July 2009 to 31 December 2012, 82 children out of 18 815 presented with heart disease, which was a prevalence of 4.3/1 000. There was a female predominance, with a ratio of 1.2. The most frequent presenting conditions were dyspnoea at 47.5%, followed by heart murmurs at 35.3%, and congestive heart failure at 13.4%. Congenital heart diseases were the most frequent, representing 69.5% of the cases, followed by acquired heart diseases at 29.3%, and mixed-type cases at 1.2%. The most frequently encountered congenital heart diseases were ventricular septal defect (24.4%), followed by atrioventricular septal defect (12.2%), tetralogy of Fallot (9.8%) and patent ductus arteriosus (7.3%). Acquired heart disease was represented by rheumatic heart disease, found in 25.6% of the cases, and tuberculous pericarditis in 3.7%. The mortality rate was high, with 20 children dying (24.4%) during the study period. Only 13 out of 82 patients (15.9%) were operable and surgery was carried out in France, courtesy of the association Humanitarian Mécénat Chirurgie Cardiaque.

Infantile heart diseases were therefore not very frequent in the paediatric unit of Louga Regional Hospital. However, congenital heart disease was more frequent than acquired heart disease, with a high mortality rate. Access to surgery remains limited.

## Resumé

La prévalence des cardiopathies infantiles en Afrique sub-Saharienne est estimée à environ 8 pour mille naissances vivantes pour les cardiopathies congénitales et au moins 1 à 14 pour mille pour les cardiopathies rhumatismales.^1^ La prise en charge de ces cardiopathies infantiles dans les pays d’Afrique au sud du Sahara et au Sénégal en particulier posent encore d’énormes difficultés de diagnostic et d’accès au traitement notamment chirurgical contribuant ainsi à une augmentation de la mortalité et de la morbidité infantile.^1^,^2^ Les objectifs de ce travail étaient de déterminer la prévalence des cardiopathies observées en milieu pédiatrique au Centre Hospitalier Régional (CHR) de Louga, de décrire les différents types observés et de rapporter leur évolution à court terme.

## Methodes

La présente étude est réalisée à Louga situé à environ 200 km au nord de la capitale, dans le service de pédiatrie du Centre Hospitalier Régional (CHR). Ce centre est un hôpital de référence au niveau régional situé en zone semi désertique, avec des ressources humaines et matérielles très limitées polarisant une population estimée à environ 960 621 habitants en 2012.3 Il s’agit d’une étude rétrospective portant sur les enfants de 0–17 ans vus en consultation externe ou hospitalisés dans le service de pédiatrie du CHR de Louga du 1 Juillet 2009 au 31 Décembre 2012.

La consultation externe est effectuée par un pédiatre ou par un infirmier. Cependant tous les cas suspects de cardiopathies vus par l’infirmier sont examinés ultérieurement par le pédiatre et bénéficiaient ainsi d’un examen clinique complet. Les patients hospitalisés sont tous vus par le pédiatre. Une cardiopathie était suspectée devant les symptômes d’appel suivants: la cyanose, la bronchite à répétition, un souffle cardiaque de caractère organique, une insuffisance cardiaque, une cardiomégalie à la radiographie du thorax. Les données des consultations externes étaient recherchées à partir des registres de consultation et celles des malades hospitalisés à partir des dossiers patients. Les doublons étant recherchés à partir de l’âge, du sexe, du lieu de résidence et du type de cardiopathie et supprimés.

**Figure 1.**
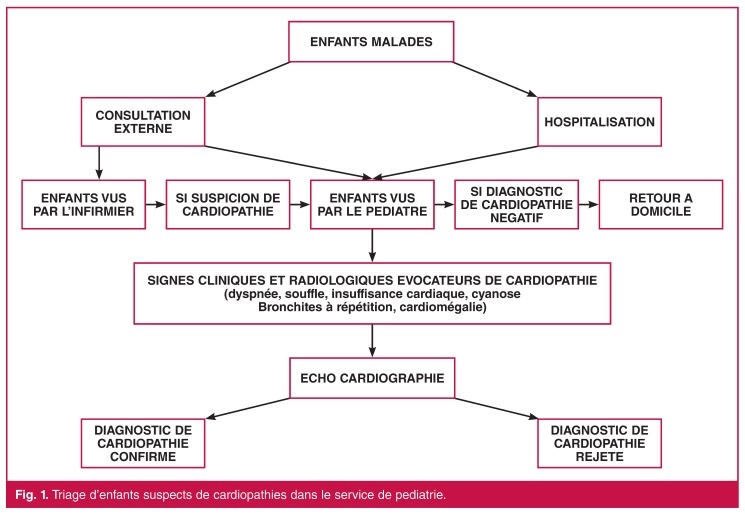
Triage d’enfants suspects de cardiopathies dans le service de pediatrie.

Une échographie cardiaque n’était prescrite qu’aux enfants présentant ces signes évocateurs d’une cardiopathie (Fig. 1). L’examen d’échographie cardiaque était effectué par un cardiologue sur un appareil HP SONOS 100 munie d’une sonde phased array de 2–4 MHz de fréquence et des modes Doppler pulsé, continue et couleur.

Le diagnostic de cardiopathie n’était retenu qu’après confirmation à l’échocardiographie. Les autres paramètres suivants ont été analysés: l’âge, le sexe, la notion de consanguinité, le niveau socio-économique, les circonstances de découverte, le type de cardiopathie acquise ou congénitale, le type de traitement médical ou chirurgical et les modalités évolutives.

Tous les parents d’enfants présentant une cardiopathie étaient informés du diagnostic et ceux ayant nécessités d’une prise en charge chirurgicale avaient donnés leur consentement préalable.

Les données recueillies ont été traitées et analysés par le logiciel Epi Info version 3.5.4.

## Resultats

Quatre-vingt-dix enfants sur les 18 815 vus durant la période d’étude présentaient des signes évocateurs d’une cardiopathie. Cinquante ont été recensé en consultation externe et 40 en hospitalisation. Ils ont tous bénéficié d’une prescription d’une échographie cardiaque, mais seuls 87 ont bénéficié de cette exploration, les parents de trois enfants ne se sont pas présenté. Cinq échographies étaient normales et 82 cardiopathies ont été confirmées soit une prévalence de 4.3/1 000.

L’âge moyen de découverte des cardiopathies congénitales est 8 ans 6 mois (extrêmes 1 mois et 15 ans) et celui des cardiopathies rhumatismaux 9 ans 5 mois (extrêmes 4ans et 15 ans). On note une prédominance du sexe féminin avec un sexe ratio de 1.2.

Le niveau socio-économique des parents est estimé moyen dans 62.2% des cas et plus de la moitié des patients (52.4%) provenaient d’un milieu urbain. Les mariages consanguins sont retrouvés chez presque tous les parents des patients (92.7%).

**Figure 2.**
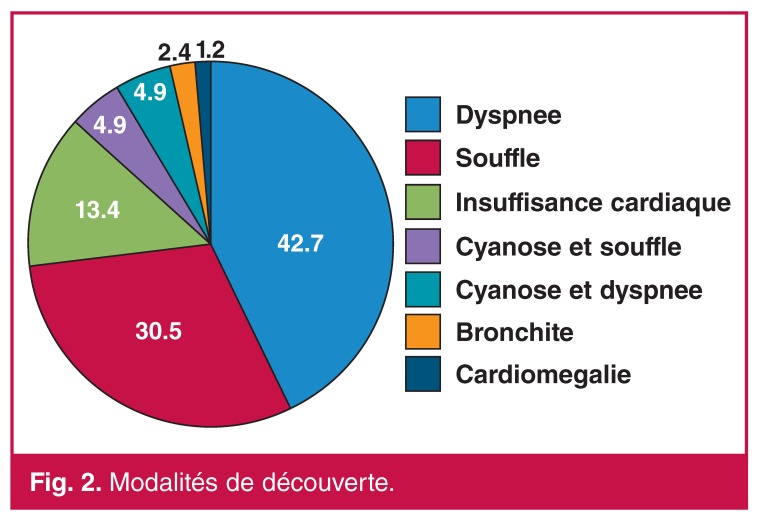
Modalités de découverte.

Les circonstances de découverte les plus fréquentes sont la dyspnée (47.5%), le souffle cardiaque (35.3%), l’insuffisance cardiaque congestive (13.4%) et la cyanose (9.7%) (Fig. 2).

Les cardiopathies congénitales sont plus fréquentes (69.5%) que les cardiopathies acquises (29.3%) (Tableau 1 et Tableau 2). Les cardiopathies congénitales sont les plus fréquentes 69.5% que les cardiopathies rhumatismales 25.6%. (Tableau 1 et Tableau 2).

**Tableau 1:** Répartition selon le type de cardiopathie

*Type*	*Nombre*	*Pourcentage*
Acquise		
Non-rhumatismale (péricardite tuberculeuse)	3	3.7
Rhumatismale	21	25.6
Congénitale	57	69.5
Mixte (congénitale et acquise: CIV, IA + IM)	1	1.2
Total	82	100

CIV = communication inter ventriculaire; IA = insuffisance aortique; IM = insuffisance mitrale.

**Tableau 2:** Répartition des différentes cardiopathies

*Diagnostics*	*Nombre (n = 82)*	*Pourcentages*
Cardiopathies congénitales		
CIV	20	24.4
CAV	10	12.2
Tétralogie de Fallot	8	9.8
PCA	6	7.3
CIA	3	3.7
APSO	2	2.4
HTAP primitive	2	2.4
CMD congénitale	2	2.4
VDDI	1	1.2
Oreillette unique	1	1.2
TGV avec CIV	1	1.2
Sténose pulmonaire	1	1.2
Cardiopathies acquises		
IM	10	12.2
IM + IA	9	11
RM	2	2.4
Péricardites tuberculeuses	3	3.7
Cardiopathies mixtes		
CIV + IM	1	1.2
Total	82	100

CIV = communication inter ventriculaire; CIA = communication inter auriculaire; CAV = canal atrioventriculaire; VDDI = ventricule droit à double issu; APSO = atrésie pulmonaire à septum ouvert; PCA = persistance du canal artériel; TGV = transposition des gros vaisseaux; CMD = cardiomyopathie dilatée; HTAP = hypertension artérielle primitive; IM = insuffisance mitrale; IA = insuffisance aortique; RM = rétrécissement mitral.

La mortalité est élevée avec 20 enfants décédés (24.4%) pendant la période d’étude. Il s’agissait de 11 cas de valvulopathies rhumatismales sévère au stade d’insuffisance cardiaque réfractaire, huit cas de cardiopathie congénitale et un cas d’un décès post opératoire précoce d’une chirurgie correctrice de tétralogie de Fallot. Treize enfants sur 82 (15.9%) présentant une indication opératoire ont été opérés en France grâce à une prise en charge de l’association humanitaire Mécénat Chirurgie Cardiaque. Cependant sept enfants présentaient une indication opératoire dépassée du fait d’une hypertension artérielle pulmonaire sévère au stade d’Eisenmenger compliquant une cardiopathie congénitale avec shunt gauche-droite sont régulièrement suivi.

Six patients (7.3%) on été transférés: cinq présentant une persistance de canal artériel (PCA) ont été transférés vers le centre de chirurgie thoracique et cardiovasculaire de Dakar pour une prise en charge chirurgicale et sont sur la liste d’attente. le dernier a été référé dans un autre hôpital proche de son lieu de résidence; 15 enfants (18.3%) sont perdus de vue.

## Discussion

Cette étude montre la faible prévalence des cardiopathies infantiles dans le service. Nous notons un diagnostic tardif des cardiopathies congénitales avec un âge moyen de 8 ans témoignant de la faible performance de nos structures sanitaires notamment l’extrême rareté en spécialiste dans les régions.^4^ Cet âge est plus élevé que dans la série de Diop à Dakar,^5^ de Mpemba à Brazzaville au Congo^6^ et Chaabouni à Sfax en Tunisie^7^ qui trouvent respectivement 6.8, 6.3 et 5 ans. L’âge des cardiopathies congénitales est cependant légèrement plus bas que dans une série de cas opérés à Dakar.^8^

L’âge moyen de découverte des cardiopathies rhumatismales est de 9 ans 5 mois, plus bas que dans la série chirurgicale de Ciss à Dakar.^9^ Les cardiopathies congénitales étaient plus fréquentes que les cardiopathies rhumatismales pouvant s’expliquer par la plus grande incidence des malformations cardiaques comparée aux cardiopathies rhumatismales. Ceci a été également retrouvé par Ba en Mauritanie^10^ et Brousse au Sénégal;^11^ 92.7% des enfants de l’étude sont issue de mariage consanguins. Cet aspect a été noté comme un important facteur contributif de malformation cardiaque.^12^

Le pronostic est défavorable dans notre contexte avec un taux de mortalité de 24.4% dépassant les 20% retrouvés à l’Hôpital Principal de Dakar en 1997 par Brousse11 et s’expliquant par le retard diagnostic et l’accès difficile à la chirurgie cardiaque. Il est à observer que si les cardiopathies se caractérisent par leur faible prévalence hospitalière elles représentent une des principales causes de morbidité et de mortalité chez l’enfant dans les pays en développement.^13^

Le taux de perdus de vue de 18.3% est plus élevé que celui retrouvé dans la série similaire au Sénégal de Brousse.^11^ Il pourrait s’expliquer par le caractère asymptomatique de certaines cardiopathies notamment congénitales et l’amélioration clinique des enfants opérés. Seul 15.9% des enfants ont pu être opéré. Ce faible taux d’accès au traitement chirurgical s’explique par plusieurs facteurs notamment l’impossibilité actuelle d’opérer des enfants de moins de 15 kg et les enfants présentant une hypertension artérielle pulmonaire avec une pression systolique supérieure à 50 mmHg.^8^

Dans ce contexte, les associations humanitaires comme ‘Mécénat Chirurgie cardiaque enfants du monde’, offrant une prise en charge jouent encore un rôle très important. Cependant il y a un intérêt indiscutable à développer la chirurgie cardiaque localement.^5^,^7^

## Limites de l’etude

Il s’agit d’une étude rétrospective qui a l’inconvénient de ne pas fournir toutes les données souhaitées notamment la Spo2 et les aspects électrocardiographiques. Le diagnostic de cardiopathie fait par un infirmier peut constituer un facteur limitant dans la mesure où une cardiopathie peu grave ou asymptomatique n’est pas détectée par ce dernier conduisant au sous diagnostic et à la sous estimation de la prévalence. Par ailleurs les nouveau-nés porteurs de cardiopathies congénitales sévères décédés peu après leur naissance ne sont pas pris en compte.

Il s’agit d’une étude hospitalière dans un contexte où l’accessibilité aux soins est limitée. Le seul moyen paraclinique de confirmation de cardiopathie était un échographe peu performant. Les autres moyens à savoir le cathétérisme cardiaque et l’imagerie résonnance magnétique qui aurait pu apporter un diagnostic précis ou limiter les faux négatifs sont indisponibles sur place et hors de portée des populations. Eu égard à la nature hospitalière de l’étude et aux facteurs limitant précités, les données ne peuvent être extrapolées à la population générale mais donnent une idée de l’existence du problème qui pourrait être mieux diagnostiqué par d’autres études.

## Conclusion

Cette étude bien que comportant des limites montre que les cardiopathies infantiles sont peu fréquentes au service de pédiatrie du CHR de Louga avec une prévalence de 4.3 pour 1 000. Les formes congénitales sont plus fréquentes que les formes acquises. L’accès à la chirurgie cardiaque est faible et la mortalité est élevée. II est donc nécessaire de mettre l’accent sur le dépistage précoce de cardiopathies infantiles et d’améliorer leur accès à la chirurgie cardiaque localement.

## References

[1] Zuhlke L, Mirabel M, Marijon E (2013). Congenital heart disease and rheumatic heart disease in Africa: recent advances and currents priorities.. Heart.

[2] Mocumbi AO, Lameira E, Yaksh A (2011). Challenges on the management of congenital heart disease in developing countries.. Int J Cardiol.

[3] (2010). Situation économique et Sociale de la région de Louga.

[4] (2009). Profil pays en ressources humaines pour la santé du Sénégal.. Mars.

[5] Diop IB, Ndiaye M, Ba SA, Sarr M, Kane A, Hane L, Sow D, Ba K, Diack B, Diouf SM, Fall M (1996). Congenital heart disease surgery in Senegal. Indications, evaluation and perspectives.. Dakar Med.

[6] M’Pemba Loufoua Lemay AB, Johnson EA, Nzingoula (2005). Les cardiopathies congénitales observées dans le service de pédiatrie Grands Enfants du CHU de Brazzaville à propos de 73 cas: Aspects épidémiologiques. Médecine d’Afrique Noire.

[7] Chaabouni M, Kamoun T, Mekki N, Mahfoudh A, Karray A, Daoud M, Triki A (1999). Aspects épidémiologiques et évolutifs des cardiopathies congénitales dans le service de pédiatrie de Sfax: A propos de 123 cas.. Tunisie Médicale.

[8] Fall ML, Leye PA, Ba PA, Bah MD, Ndiaye PI, Ciss AG, Sene E, Kane K, Kane O, Diouf E (2012). La prise en charge péri opératoire des cardiopathies congénitales au Sénégal.. Rev Afr Anesth Méd Urg.

[9] Ciss AG, Diarra O, Dieng PA, N’diaye A, Ba PS, Touré A, Diatta S, Beye SA, Kane O, Diop IB, N’diaye M (2009). La plastie mitrale sur valve rhumatismale chez l’enfant au Sénégal: 100 observations.. Med Trop.

[10] Ba ML, Kane FB (2000). Etude préliminaire des cardiopathies chez l’enfant mauritanien.. Médecine d’Afrique Noire.

[11] Brousse V, Imbert P, Mbaye P, Kieffer K, Thiam M, Ka AS, Gerardin P, Sidi D (2003). Evaluation au Senegal du devenir des enfants transférés pour chirurgie cardiaque.. Méd Trop.

[12] Yunis K, Mumtaz G, Bitar F, Chamsedine F, Kassar M, Rashkidi J (2006). Consanguineous marriage and congenital heart defects: A case–control study in the neonatal period.. Am J Med Genet A.

[13] Deen J, Vos T, Huttly SRA, Tulloch J (1999). Traumatismes et maladies non transmissibles: des pathologies émergentes chez les enfants des pays en développement.. Bull WHO.

